# Preparation and Characterization of Materials Based on Graphene Oxide Functionalized with Fe, Mn, Ni, and Cu Oxides and Their Testing for the Removal of Water Pollutants

**DOI:** 10.3390/ma18122735

**Published:** 2025-06-11

**Authors:** Ocsana Opriș, Adina Stegarescu, Ildiko Lung, Alin Sebastian Porav, Irina Kacso, Gheorghe Borodi, Cristian Leoștean, Ovidiu Pană, Maria-Loredana Soran

**Affiliations:** National Institute for Research and Development of Isotopic and Molecular Technologies, 67-103 Donat, 400293 Cluj-Napoca, Romaniasebastian.porav@itim-cj.ro (A.S.P.); ovidiu.pana@itim-cj.ro (O.P.); loredana.soran@itim-cj.ro (M.-L.S.)

**Keywords:** graphene oxide, metal oxides, pollutant, pesticide, drug, adsorption, decontamination

## Abstract

Nanotechnology has emerged as a highly focused field of research due to the unique properties of nanometric materials, particularly their large specific surface areas and excellent adsorption capabilities. This study investigated the synthesis of materials based on graphene oxide (GO) functionalized with different metal oxides (MnO_2_, Fe_3_O_4_, CuO, NiO), with potential applications in water decontamination. The morphological, structural, and compositional properties of these nanocomposites were extensively characterized using different experimental techniques, including X-ray diffraction (XRD), transmission electron microscopy (TEM), Fourier transform infrared spectroscopy (FTIR), X-ray photoelectron spectroscopy (XPS), and vibrating sample magnetometry (VSM) for magnetic property evaluation. Preliminary adsorption tests were performed for the removal of pesticides and drugs from aqueous solutions. The synthesized materials demonstrated a higher affinity for selected pesticides compared to drugs. The best removal efficiencies were 98.59% for cymoxanil, 97.93% for triadimefon, 63.33% for sulfamethoxazole, and 99.59% for diclofenac. The results indicate that the functionalization of GO with metal oxides modifies the material’s structure, increasing its potential for environmental applications such as water purification.

## 1. Introduction

Water pollution continues to be a major global issue, threatening both human health and environmental sustainability. The presence of different organic and inorganic contaminants in water sources presents significant risks due to their high toxicity, persistence, and concentrations. These pollutants can have wide-ranging effects on ecosystems, public health, and economic stability [1]. Effective solutions, methods, and technologies are being explored to address the issues caused by these pollutants. An intensively addressed field for this purpose is nanotechnology because nanometric materials have large specific surfaces with good adsorption properties. Nanoparticles, nanopowders, and nanomembranes are commonly used for the efficient chemical or biological removal of pollutants from contaminated wastewater.

Graphene is a two-dimensional, very well-known, and valuable material made up of a single layer of carbon atoms arranged in a hexagonal lattice. It has attracted considerable interest due to its important properties, which include exceptional electrical and thermal conductivity, excellent mechanical strength, and an extensive surface area [2,3,4]. However, even with these important advantages, pure graphene faces several limitations. It tends to aggregate due to strong van der Waals forces, exhibits low dispersibility in solvents, and lacks a band gap—factors that restrict its use in multiple applications [5,6]. Moreover, graphene’s chemical stability can be affected under specific environmental conditions, reducing its long-term performance [7].

To manage these limitations, graphene is frequently combined with inorganic nanoparticles, such as metal oxides (MOx) or metal sulfides, resulting in graphene-based composites with increased functionalities [8,9]. These composites showed improved dispersion, more efficient charge separation, and increased mechanical and thermal stability [10,11,12]. Therefore, graphene–inorganic nanocomposites offer a promising approach to enhancing the functionalities of graphene for advanced technological applications [7].

MOx presented great potential in the field of the adsorption of pollutants [13,14]. Thus, MOx can play important roles in the areas of science and technology. Among them, CuO has been exploited in the last few years for applications in catalysts, semiconductors, sensors, or field transistors. CuO nanoparticles alone can also act as an effective adsorbent for water contaminants. Furthermore, these nanoparticles are also used to modify adsorbents such as activated carbon, silica, graphene, etc., which act as support for the nanoparticles to remove pollutants. The presence of CuO nanoparticles on activated carbon improves chemical adsorption [15]. MnO_2_ is known to present good catalytic and absorption properties, and research has started to focus on wastewater treatment by using nanostructured MnO_2_ [16]. NiO is a widely studied material with applications in lithium-ion batteries, catalysis, and in adsorption processes. Its applications are determined by the surface area, the trapping of generated charge carriers, or by the interplay between its optical bandgap energy. Despite its low cost, it is not an efficient photocatalyst alone due to its wide bandgap and quick recombination of photogenerated charge carriers. NiO has also been applied in water depollution efforts due to its good affinity towards dyes, but its selective adsorption property against dyes or antibiotics is still much less explored [17]. These deficits, observed in the pristine MOx, can be easily improved by combining these MOx with carbon-based nanostructures [18]. Based on the literature, it is possible to say that carbon nanostructures with MOx represent a new generation of materials that have new properties due to their interaction between the carbon nanostructures properties and MOx properties. Due to these interactions, new properties (e.g., large surface areas, chemically inert surfaces, uniform structures, multiple adsorption sites) can be generated. Along with these properties, by including some magnetic nanoparticles in the materials (e.g., iron-based nanoparticles), we have the advantage that these materials can be recovered easily with a magnet and reused. Alicanoglu and Sponza, in 2015, demonstrated that magnetite nanoparticles have the potential to remove drugs from residual waters [19].

Graphene oxide (GO) is one of the most used graphene derivatives [20]. GO is an oxidized form of graphene that features oxygen-containing functional groups, including carboxyl, hydroxyl, and epoxy groups on its surface [21,22]. These functional groups give hydrophilic properties to GO, enabling it to form stable aqueous suspensions, which facilitates processing and functionalization.

The combination of GO with MOx has demonstrated synergistic properties that improve performance across a range of applications [7], particularly in the environmental remediation, sensing, and biomedical domains. An important application of these materials is water purification. In this case, graphene-based materials function as highly effective adsorbents for the removal of pollutants such as heavy metals, organic dyes, and pharmaceutical residues. These promising results were obtained due to their large surface area and strong adsorption capabilities [23,24]. Furthermore, its excellent photocatalytic properties allow the degradation of organic contaminants under light irradiation, positioning them as highly promising tools for advanced wastewater treatment technologies [25,26,27].

Pesticides are a major class of persistent organic pollutants that are commonly released into environmental water sources because of agricultural practices [28]. Because pesticides a pose high toxicity risk, the World Health Organization (WHO) and the European Union (EU) have established the maximum allowable concentrations in water: 0.5 μg L^−1^ for surface water and 0.1 μg L^−1^ for drinking water [29]. Therefore, monitoring and development of removal methods for pesticides in water sources is essential for food safety and human health protection [30].

The removal of pesticides can be facilitated using nanoparticles [31]. Nanoparticle-assisted photocatalysis represents an efficient approach for the degradation of various pesticide compounds. Additionally, nanotechnology based on green chemistry principles has been shown to enhance the decomposition rates of numerous environmental pollutants, including pesticides [32]. Nanomaterials, such as TiO_2_, ZnO, and GO nanosheets, have been demonstrated to have strong photocatalytic performance, to rapidly degrade pesticides (atrazine and chlorpyrifos). Compared to conventional techniques, this approach offers significant advantages, including greater efficiency, lower costs, and the absence of secondary pollution, making it well-suited for applications such as drinking water purification, wastewater treatment, agricultural runoff control, and surface water remediation [33]. GO synthesized with silver nanoparticles using *Cucurbita maxima* (pumpkin) leaves effectively degraded chlorpyrifos under the presence of sunlight [34]. A ZnO/α-Fe_2_O_3_ nano photocatalyst was demonstrated to remove 89% of carbamate pesticides under optimal pH and temperature conditions [35]. The developed GO–TiO_2_ nanocomposite achieved a degradation efficiency exceeding 80% of organophosphate pesticides, specifically dichlorvos and malathion [36].

Antibiotics are a significant group of emerging environmental contaminants. Among them, sulfonamide antibiotics are extensively used both for disease treatment and preventive measures. Due to their anionic nature, sulfonamides are not efficiently removed by conventional wastewater treatment processes, thus increasing attention has been directed toward their presence in natural water bodies and water treatment systems. Research into nanomaterial-based composites for water purification is ongoing, but it remains in an early developmental stage. Designing cost-effective synthesis methods for these nanomaterials continues to be a significant challenge [37,38,39,40,41]. Materials based on graphene have shown effectiveness in adsorbing antibiotics from wastewater, leading to a significant reduction in their concentration. The high surface area and customizable properties of these materials improve their removal efficiency, positioning them as promising options for advanced water treatment technologies [42]. The CuO–GO composite demonstrated high antibiotic removal efficiencies, with adsorption capacities of 405 mg g^−1^ for amoxicillin and 552 mg g^−1^ for tetracycline. The developed material showed an 80% regeneration efficiency and maintained 82% of its adsorption capacity after five reuse cycles [43].

Diclofenac is a very well-known and often-used pharmaceutical, and is an environmental contaminant that enters water bodies due to its widespread use as a non-steroidal anti-inflammatory drug [44,45]. The global consumption of diclofenac is increasing due to the growth of populations and access to healthcare in many countries [46]. Therefore, the concentration of diclofenac in the environment can be up to 110 ng L^−1^ [47], presenting potential toxicity risks to the environment and global ecosystems. Consequently, diclofenac is considered an emerging environmental contaminant, and its removal from the environment represents an important global concern [48]. GO demonstrated a removal efficiency of nearly 100% for concentrations of diclofenac ranging from 50 to 450 mg L^−1^. For these good results, the necessary GO amounts of 0.46 and 1.38 g L^−1^, respectively, were used to remove the diclofenac from an aqueous solution [49].

The present study aimed to obtain materials based on graphene oxide (GO) functionalized with transition metal oxides (MOx: MnO_2_, Fe_3_O_4_, CuO, NiO) and test their use in water decontamination. The investigation of the morphological, structural, and compositional properties of these materials was carried out using experimental techniques, such as X-ray diffraction (XRD), transmission electron microscopy (TEM), scanning electron microscopy (SEM), Fourier transform infrared spectroscopy (FTIR), X-ray photoelectron spectroscopy (XPS), and the magnetic properties of the nanocomposites were determined using the vibrating sample magnetometry (VSM) method. We hypothesized that the functionalization of the GO with selected MOx leads to improved adsorption performance for the removal of organic pollutants, particularly pesticides and drugs, from aqueous solutions. According to our knowledge, we can say that they are new combinations of materials with unique physicochemical characteristics, and these specific MOx combinations are expected to significantly increase the affinity and removal efficiency of the materials, making them suitable candidates for advanced water decontamination applications. 

## 2. Materials and Methods

### 2.1. Chemicals

Graphite powder (96–98%) was purchased from KOH-I-NOOR HARDTMUTH a.s. (České Budějovice, Czech Republic). Sulfuric acid (H_2_SO_4_, 97%) was purchased from Chomchim Chemical (Râmnicu Vâlcea, Romania). Potassium permanganate (KMnO_4_, 99%) was bought from Roth (Karlsruhe, Germany) and hydrogen peroxide (H_2_O_2_, 30%) from Chimopar Trading (Bucureşti, Romania). MnO_2_ was prepared according to Lung et al. [50]. Iron (III) chloride hexahydrate (FeCl_3_•6H_2_O, 97%), iron (II) sulfate heptahydrate (FeSO_4_•7H_2_O, 99%), potassium bromide (KBr, 99%), sulfamethoxazole (98%), sodium nitrate (NaNO_3_, 99%), and hydrochloric acid (HCl, 37%) were purchased from Sigma–Aldrich (Taufkirchen, Germany). Ammonium hydroxide (NH_4_OH, sol. 25%), nickel chloride hexahydrate (NiCl_2_•6H_2_O, 98%), L-ascorbic acid (99%), cetyltrimethylammonium bromide (CTAB, 99%), cymoxanil (99%), and triadimefon (99%) were purchased from Merck (Taufkirchen, Germany). Sodium hydroxide (NaOH, 97%) and acetonitrile graded for high-performance liquid chromatography (HPLC purity, 99.9%) was purchased from VWR Chemicals (Vienna, Austria). Diclofenac was bought from Refen (Hemofarm, Cluj-Napoca, Romania), and formic acid was purchased from Cristal R Chim (Bucureşti, Romania). In all the experiments, ultrapure water was produced by a Direct-Q 3 UV Water Purification System (Merck, Taufkirchen, Germany).

### 2.2. Materials Synthesis

#### 2.2.1. GO Synthesis

Into a 500 mL flask, 2.5 g of graphite powder (purity 96–98%) was added, which was mixed with 1.25 g of NaNO_3_ and 118 mL of H_2_SO_4_ (97%), followed by magnetic stirring at 500 rpm in an ice bath for 30 min (˂20 °C). After this step, 7.5 g of KMnO_4_ was added (in small portions) while stirring vigorously (1000 rpm). The mixture was stirred for 2 h at a temperature below 20 °C. The flask was then heated in a water bath at 35 °C and kept under stirring conditions for 30 min at this temperature, and then 115 mL of deionized water was added, heating the water bath to 98 °C and keeping it that way for 15 min. The obtained paste was diluted by adding 350 mL of deionized water. After this step, 30% H_2_O_2_ was added dropwise until a bright yellow color was obtained. The obtained suspension was filtered and washed with 800 mL of 5% HCl and 300 mL of deionized water. The precipitate was dried by lyophilization.

#### 2.2.2. GO-MnO_2_ Synthesis

To synthesize GO-MnO_2_, 0.02 g of GO was shaken for 30 min with 20 mL of bidistilled water. In parallel, 0.1 g of MnO_2_ was shaken with 25 mL of bidistilled water for 30 min. The MnO_2_ suspension was added to the GO suspension, and stirring was continued on the shaker for another 6.5 h. The precipitate formed was washed by centrifugation with 3 portions of bidistilled water and then with 3 portions of ethanol. The obtained material was dried overnight in an oven at 60 °C.

#### 2.2.3. GO-Fe_3_O_4_ Synthesis

To synthesize GO-Fe_3_O_4_, 0.06 g of GO was stirred in an ultrasonic bath for 20 min with 36.12 mL of bidistilled water, and then stirring was continued on a magnetic plate, under argon, for 30 min at 60 °C. Further, 91.8 mg of FeCl_3_•H_2_O was added to the obtained suspension, and stirring was continued for another 30 min, after which 48 mg of FeSO_4_•7H_2_O was added, and stirring was then continued for another 30 min. Finally, 18 mL of 6% NH_4_OH was added in thin drops. The reaction mixture was left to stir for another 2 h. The obtained material was washed by centrifugation with bidistilled water until a neutral pH was obtained, and then dried overnight in an oven at 60 °C.

#### 2.2.4. GO-Fe_3_O_4_-NiO Synthesis

To synthesize GO-Fe_3_O_4_-NiO, 0.02 g of GO-Fe_3_O_4_ was ultrasonicated for 30 min in 12 mL of bidistilled water. To this suspension were added freshly prepared solutions of 0.1249 g NiCl_2_•6H_2_O in 50 mL of bidistilled water, and 0.9687 g of ascorbic acid in 50 mL of bidistilled water. After adding 0.5467 g of CTAB to the previously obtained suspension, 50 mL of bidistilled water was added and the mixture was brought to a pH of 6.5 with a NaOH solution, after which it was heated to 85 °C and stirring was continued for 3 h. The sample was washed by centrifugation with bidistilled water and dried in an oven at 75 °C.

#### 2.2.5. GO-Fe_3_O_4_-CuO Synthesis

To synthesize GO-Fe_3_O_4_-CuO, 0.02 g of GO-Fe_3_O_4_ was ultrasonicated for 30 min in 12 mL of bidistilled water. To this suspension were added freshly prepared solutions of 0.1249 g of CuSO_4_•5H_2_O in 50 mL of bidistilled water, and 0.9687 g of ascorbic acid in 50 mL of bidistilled water. After adding 0.5467 g of CTAB to the previously obtained suspension, 50 mL of bidistilled water was added and the mixture was brought to a pH of 6.5 with a NaOH solution, after which it was heated to 85 °C and stirring was continued for 3 h. The sample was washed by centrifugation with bidistilled water and dried in an oven at 75 °C.

### 2.3. Materials Characterization

#### 2.3.1. Powder X-Ray Diffraction (XRD)

X-ray diffractograms were made with a Bruker D8 Advance diffractometer with a Cu tube CuKα1 radiation. The samples were scanned using the DIFFRAC Plus XRD Commander program (Karlsruhe, Germany). For monochromatizing radiation, a Ge(111) monochromator was used, and a fast LynxEye position detector (Karlsruhe, Germany) was used for the detection of diffracted radiation. Bragg–Brentano reflective geometry was used.

#### 2.3.2. Morphological Characterization

Determination of the morphology of the samples was performed by transmission electron microscopy (TEM) and reflection electron microscopy (REM) measurements using a HITACHI HD-2700 microscope (Tokyo, Japan). The microscope was coupled with an elemental X-ray diffraction detector (EDX) (Tokyo, Japan).

#### 2.3.3. FTIR Analysis

The FTIR spectroscopic analysis of the samples was performed with a JASCO FTIR-6100 spectrometer (JASCO International Co., Ltd., Tokyo, Japan) in the 4000 to 400 cm^−1^ spectral domain, with a 4 cm^−1^ resolution. For sample preparation, the KBr pellet technique was used. Each sample was dispersed in about 300 mg of anhydrous KBr and mixed in an agate mortar, then the prepared mixtures were pressed into an evacuated die. The collection and analysis of spectral data were carried out using JASCO Spectra Manager v.2 software.

#### 2.3.4. X-Ray Photoelectron Spectroscopy (XPS) Analysis

The qualitative and quantitative composition of the samples were investigated using an XPS with a custom-built SPECS spectrometer, (SPECS, Berlin, Germany). An Al anode (1486.71.6 eV) was used as the X-ray source. Sample preparation was done by casting onto a sample holder using ethanol. Argon ion sputtering was performed at 1000 V/10 mA. CasaXPS version 2.3.14 was used for spectral analysis by using the relative sensitivities, transmission factors, and electronic mean free path factors. A Shirley background was extracted from the core-level spectra.

#### 2.3.5. Vibrating-Sample Magnetometry (VSM) Analysis

The room temperature magnetic hysteresis measurements were performed using a Vibrating Sample Magnetometer produced by Cryogenic, (London, UK).

### 2.4. Preliminary Adsorption Tests

The obtained materials were preliminarily tested for the removal of pesticides (cymoxanil and triadimefon) and drugs (sulfamethoxazole and diclofenac) from aqueous solutions. In the case of pesticides, a removal method described by Lung et al. (2023) was used [51]. The initial concentration of cymoxanil was 7 mg L^−1^, brought to a pH of 4 with an HCl solution. It was combined with an adsorbent dose of 1 g L^−1^, a temperature of 20 °C, a contact time of 5 min, and stirring at 300 rpm. For the testing of the triadimefon removal from aqueous solution using the obtained materials, the experimental conditions were described by Lung et al. (2022) [52]. In this case, the initial concentration of triadimefon was 20 mg L^−1^, brought to a pH of 2 with an HCl solution, and then combined with the adsorbent dose of 1 g L^−1^, a temperature of 20 °C, a contact time of 4 min, and stirring at 300 rpm.

The conditions for removal of the selected drugs using the prepared materials followed those described by Lung et al., 2021: an initial drug concentration of 40 mg L^−1^, a pH of 2, 20 min of contact time, stirring at 300 rpm, and the same adsorbent dose (1 g L^−1^) and temperature (20 °C) [50].

All the samples were filtered using nylon syringe filters (13 mm × 0.22 µm) and analyzed using HPLC with a photodiode array detector PDA (Shimadzu LC-2010, Tokyo, Japan).

The HPLC analysis conditions used for the determination of the pesticides were as follows: column Nucleosil 100-5 C18 EC 250 × 4.6 mm (Machery-Nagel, Germany), thermostated at 40 °C, isocratic elution with the mobile phase consisting of acetonitrile (A):ultrapure water with 0.1% formic acid (B), 60:40 (*v*/*v*), a flow of 0.4 mL min^−1^, and an injection volume of 10 µL [52]. For the HPLC analysis of the drugs, the same chromatographic conditions were used, except for the composition of the mobile phase A, which consisted of 90:10 (*v*/*v*) acetonitrile:ultrapure water. A known concentration of 0.01 mg L^−1^ pesticide/drug was added to each sample, and all were analyzed in triplicates.

The removal efficiency (η %) of the selected pollutants from the aqueous solution was calculated by subtracting the pollutant concentration at time t (4, 5, or 20 min, depending on the pollutant) from the concentration at time 0. This difference was then divided by the concentration at time 0 and multiplied by 100.

## 3. Results and Discussion

### 3.1. XRD Characterization

**Figure 1:**
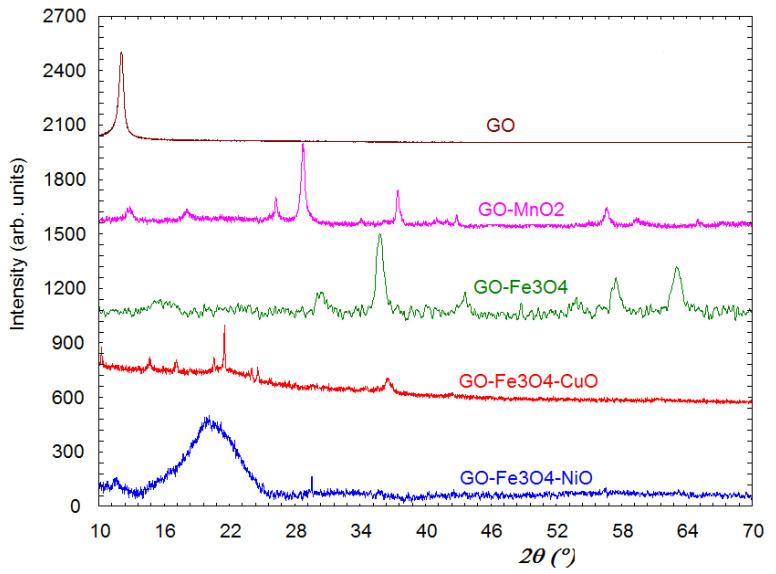
X-ray diffraction patterns of synthesized materials: GO, GO-MnO_2_, GO-Fe_3_O_4_, GO-Fe_3_O_4_-CuO, and GO-Fe_3_O_4_-NiO.

Figure 1 shows the X-ray diffractograms for GO, GO-Fe_3_O_4_, GO-Fe_3_O_4_-CuO, and GO-Fe_3_O_4_-NiO. It is observed that the diffraction line appears at the characteristic 12° of GO. From the half-width of this diffraction line, the crystallite sizes for GO were evaluated as 182Å. For GO-Fe_3_O_4_, it is observed that the diffraction peak corresponding to GO fades and moves to higher angles, namely 15.5°. There is probably a tendency for GO to transform into graphene. For this sample, all the diffraction lines characteristic of the Fe_3_O_4_ phase were present. For the GO-Fe_3_O_4_-CuO sample, the following crystalline phases were present: Cu_2_O, FeO, and Fe_2_O_3_. There is also a diffraction halo centered at an angle of 21.4° that lost some of the oxygen characteristics of GO because of its tendency to transform into graphene. GO-Fe_3_O_4_-NiO is characterized by two diffraction maxima. One of them has the low-intensity characteristics of the initial GO, and the second one has a more intense halo centered at approximately 20°, which probably indicates the existence of oxygen-depleted GO.

### 3.2. TEM Characterization

**Figure 2:**
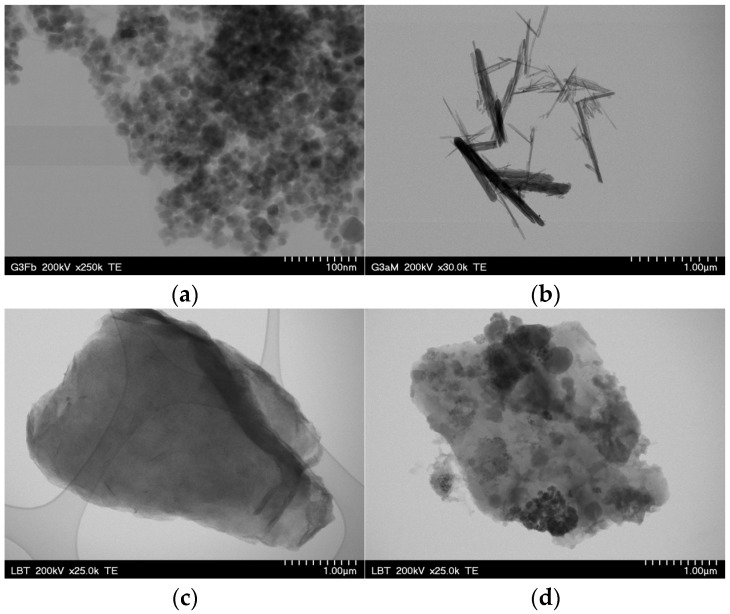
TEM images for GO-Fe_3_O_4_ (**a**), GO-MnO_2_ (**b**), GO-Fe_3_O_4_-NiO (**c**), and GO- Fe_3_O_4_-CuO (**d**).

Figure 2 shows the TEM images of the GO-Fe_3_O_4_ (a), GO-MnO_2_ (b), GO-Fe_3_O_4_-NiO (c), and GO-Fe_3_O_4_-CuO (d) nanocomposites. It is observed that the shape of the Fe_3_O_4_ nanoparticles is spherical, that of the MnO_2_ nanoparticles is acicular, and that both are up to approximately 50 nm in size. A variation in the size of the MnO_2_ needles is observed, as well as their welding. With the addition of the second phase—CuO and NiO, respectively—to the compound, the embedding of the nanoparticles in a matrix of GO nanoparticles is observed.

**Figure 3:**
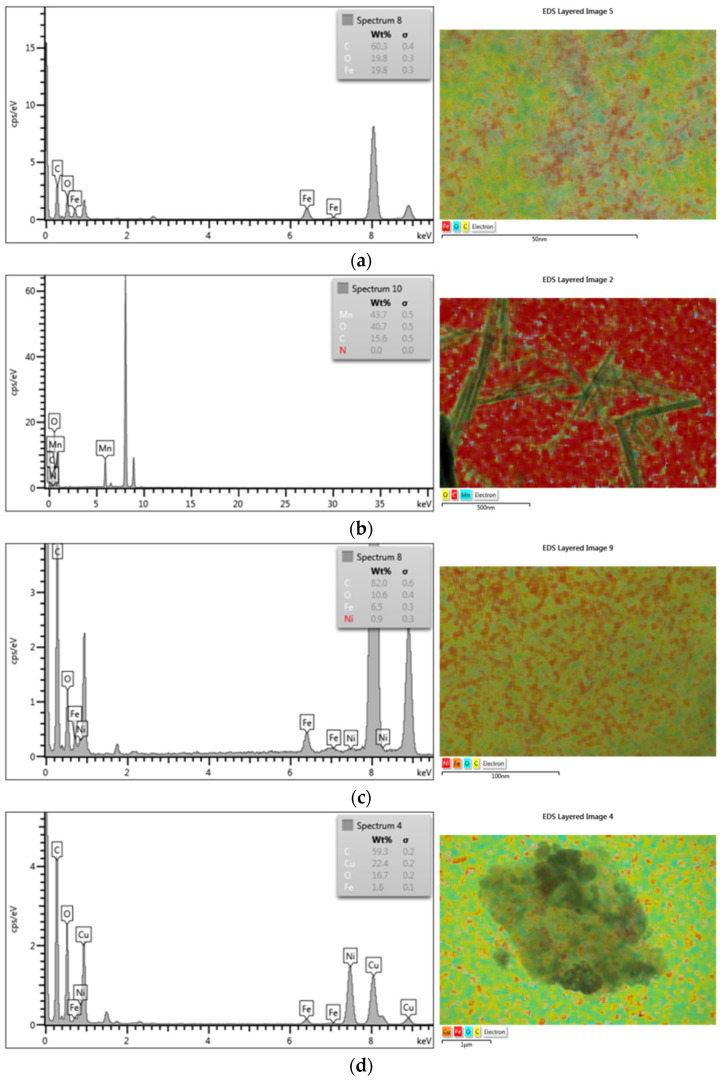
EDX spectrum and elements maps registered for the materials: GO-Fe_3_O_4_ (**a**), GO-MnO_2_ (**b**), GO-Fe_3_O_4_-NiO (**c**), and GO-Fe_3_O_4_-CuO (**d**).

Compositional determination by EDX in the marked areas indicates the presence of elements belonging to all the component phases of the nanocomposites (Figure 3). The element distribution maps show the distribution of the elements and, implicitly, the component phases in the chosen sample. The presence of all the elements of both metallic and organic component phases was found.

### 3.3. FTIR Characterization

**Figure 4:**
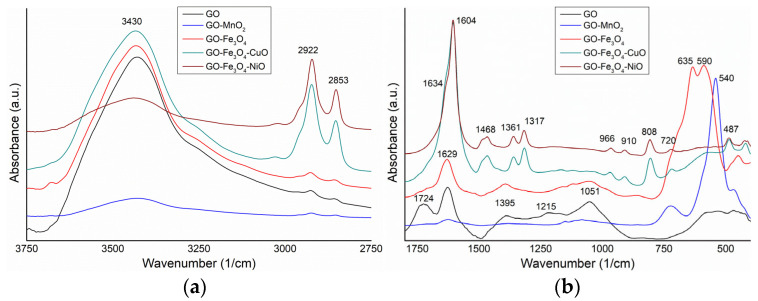
FTIR spectra of GO, GO-MnO_2_, GO-Fe_3_O_4_, GO-Fe_3_O_4_-CuO, and GO-Fe_3_O_4_-NiO in the 3750–2750 cm^−1^ spectral domain (**a**), and 1750–400 cm^−1^ spectral domain (**b**).

The spectra of the analyzed samples are comparatively presented in Figure 4a,b. The characteristic vibrational bands of GO were identified as follows: 3430 cm^−1^ (-OH stretching vibrations), 2925 and 2855 cm^−1^ (-CH_2_ and -CH_3_ groups asymmetric and symmetric stretching), 1724 and 1627 cm^−1^ (-C=O and aromatic -C=C- stretching), 1395 and 1215 cm^−1^ (C-OH stretching and -O-H deformation), 1051 (C-O bonds stretching) and broadband with a weak intensity of 690–400 cm^−1^ [53,54,55].

The spectrum of the GO-MnO_2_ sample presents the following vibrational bands: 3428 cm^−1^ (O-H stretching), 2923 and 2854 cm^−1^ (C-H stretching), 1705 and 1624 cm^−1^ (-C=O and aromatic C=C stretching), 1385 cm^−1^ (O-H deformation), 1151 and 1082 cm^−1^ (C-O stretching), 724 cm^−1^ (Fe-O stretching), and a strong band at 540 cm^−1^ (Mn-O stretching from MnO_2_) [56].

In the spectrum of the GO-Fe_3_O_4_ sample, the following vibrational bands can be identified: 3430 cm^−1^ (O-H stretching); 2922 and 2853 cm^−1^ (C-H stretching); 1623 cm^−1^ (aromatic C=C stretching); 1392 cm^−1^ (O-H deformation); 1160, 1118, and 1053 cm^−1^ (C-O stretching); 635 and 590 cm^−1^ (Fe-O stretching from Fe_2_O_3_, and Fe-O stretching from Fe_3_O_4_); and 450 cm^−1^ (Fe-O stretching from Fe_2_O_3_) [57].

The spectrum of the GO-Fe_3_O_4_-CuO sample shows the following vibrational bands: 3433 cm^−1^ (O-H stretching); 2919 and 2849 cm^−1^ (C-H stretching); 1604 cm^−1^ (C=C stretching); 1466, 1361 and 1317 cm^−1^ (O-H deformation); a broad and weak band between 1250 and 1000 cm^−1^ (C-OH and C-O stretching); 969, 908, 805, and 719 cm^−1^ (Fe-O stretching from Fe_2_O_3_); a broad band between 670 and 530 cm^−1^ with maxima at 561 cm^−1^ (Fe-O stretching from Fe_3_O_4_) [57]; 486 cm^−1^ (Cu-O bond stretching from CuO) [58]; and 420 cm^−1^ (Fe-O stretching of Fe_2_O_3_).

In the spectrum of the GO-Fe_3_O_4_-NiO sample, vibration bands were identified at the following wavenumbers: 3438 cm^−1^ (O-H stretching); 2922 and 2853 cm^−1^ (C-H stretching); 1631 and 1604 cm^−1^ (-C=O and aromatic C=C stretching); 1482, 1468, and 1361 cm^−1^ (O-H stretching); 1317, 966, 910, 808, and 720 cm^−1^ (Fe-O stretching from Fe_2_O_3_); a weak and broad band between 680 and 500 cm^−1^, with small maxima at 610, 580, and 541 cm^−1^ (Ni-O-H and Fe-O stretching from Fe_3_O_4_); and 486, 421, and 412 cm^−1^ (Ni-O stretching) [57,59].

Using comparative analysis of the spectra, a weak intensity band of O-H stretching vibrations was observed at 3430 cm^−1^ for the GO-MnO_2_ and GO-Fe_3_O_4_-NiO samples. Also, the stretching vibrations of C-H bonds between 2922 and 2853 cm^−1^, and aromatic C=C stretching from 1604 cm^−1^, have a much higher intensity in the case of GO-Fe_3_O_4_-CuO and GO-Fe_3_O_4_-NiO compared to the other samples. It was observed that the intensity of the vibrations of Fe-O bonds was greatly reduced as a result of the addition of Cu-O and Ni-O.

### 3.4. XPS Characterization

**Figure 5:**
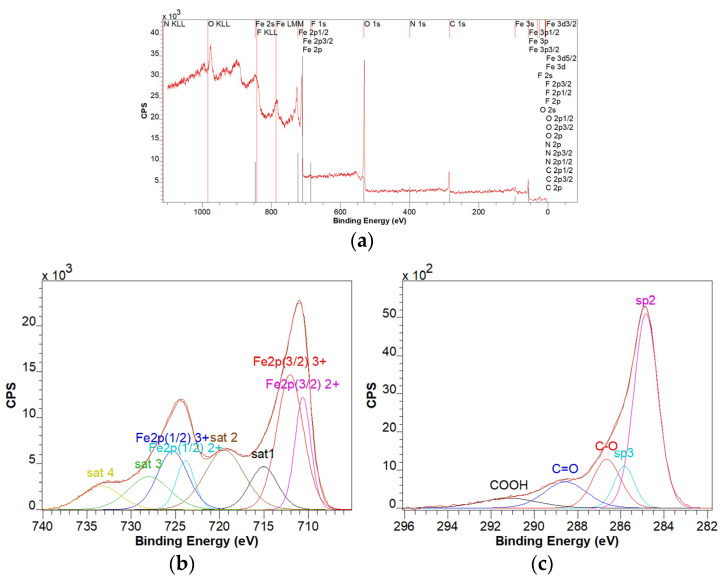
XPS survey spectrum of the GO-Fe_3_O_4_ sample (**a**), XPS spectrum deconvolution corresponding to the Fe 2p line (**b**), and XPS spectrum deconvolution corresponding to the C 1s line (**c**).

For the GO-Fe_3_O_4_ sample, from the analysis of the survey spectrum (Figure 5a), it was found that the Fe, F, N, O, and C elements are present. The multiplet structure of the Fe 2p spectrum (Figure 5b) is characteristic of the 2+ and 3+ oxidation states corresponding to the Fe_3_O_4_ compound. The C 1s spectrum (Figure 5c) deconvolution was done by considering the specific lines for the graphene oxide C=C sp2, C-C sp3, C-O/C-OH, C=O, and COOH [60]. The sp3/sp2 ratio was 0.18. One possible source of fluorine contamination was the Teflon stirrer.

**Figure 6:**
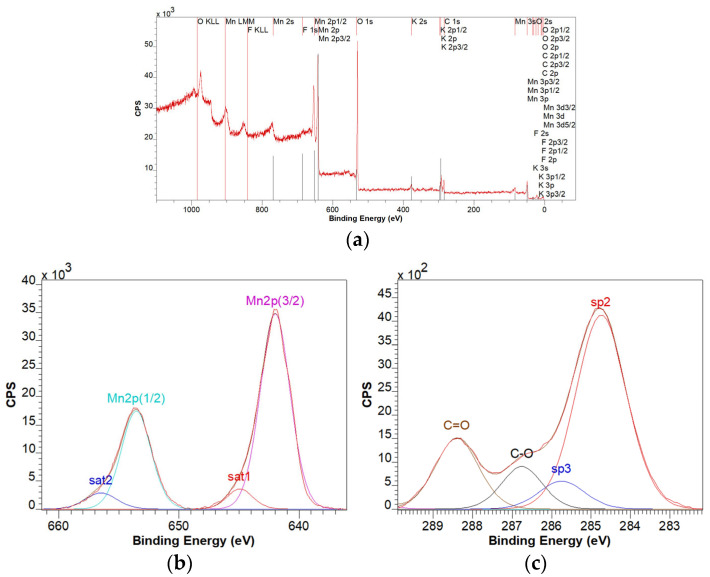
XPS survey spectrum of the GO-MnO_2_ sample (**a**), XPS spectrum deconvolution corresponding to the Mn 2p line (**b**), and XPS spectrum deconvolution corresponding to the C 1s line (**c**).

For the GO-MnO_2_ sample, from the analysis of the survey spectrum (Figure 6a), it was found that the elements Mn, F, K, O, and C are present. The multiplet structure of the Mn 2p spectrum (Figure 6b) is characteristic of the 4+ oxidation state, corresponding to the MnO_2_ compound. The analysis of the C1s spectrum reveals the specific lines for the graphene oxide with a sp3/sp2 ratio of 0.13.

**Figure 7:**
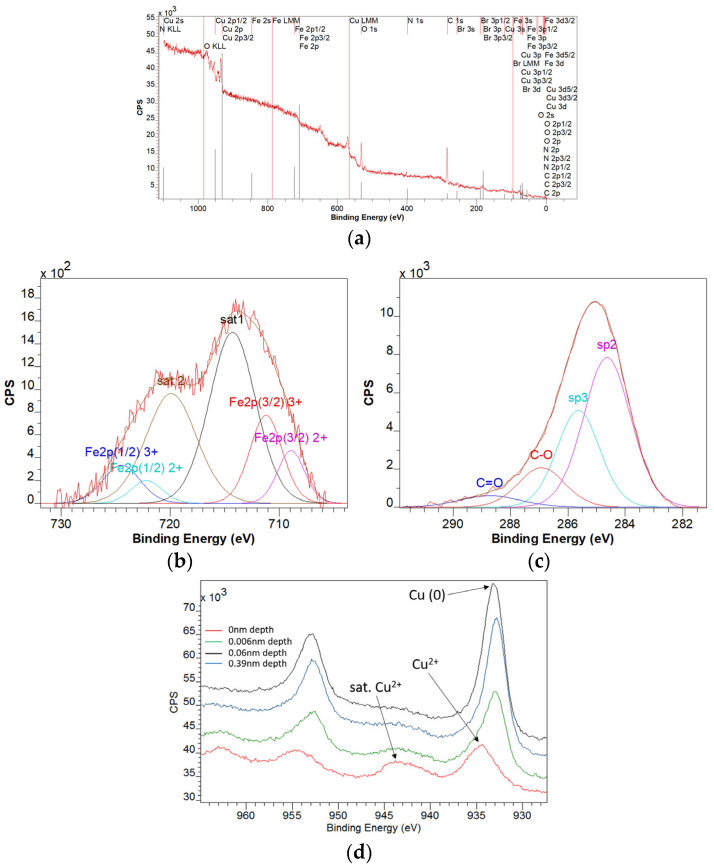
XPS survey spectrum of the GO-Fe_3_O_4_-CuO sample (**a**), XPS spectrum deconvolution corresponding to the Fe 2p line (**b**), XPS spectrum deconvolution corresponding to the C 1s line (**c**), and XPS spectrum deconvolution corresponding to the Cu 2p line at different depths up to 0.39 nm (**d**).

For the GO-Fe_3_O_4_-CuO sample, from the survey spectrum analysis (Figure 7a), it was found that the elements Fe, Cu, Br, N, O, and C are present. The multiplet structure of the Fe 2p spectrum (Figure 7b) is characteristic of the 2+ and 3+ oxidation states corresponding to the Fe_3_O_4_ compound. The depth profile analysis indicates that Fe is only in the oxidized state. The analysis of the C1s spectrum (Figure 7c) reveals the specific lines for the graphene oxide with a sp3/sp2 ratio of 0.58. The multiplet structure of the Cu 2p spectrum (Figure 7d) is characteristic of the 2+ and 0 oxidation states. The depth profile analysis indicates that the Cu oxidation state is (0), and there was a superficial oxide layer on the surface. At a depth of 0.39 nm, the ratio of Cu(0)/Cu(ox) = 5.39. Analysis of the depth profile of the Br 3p line indicated a homogeneous distribution of Br in the sample. Residual Br comes from CTAB precursor.

**Figure 8:**
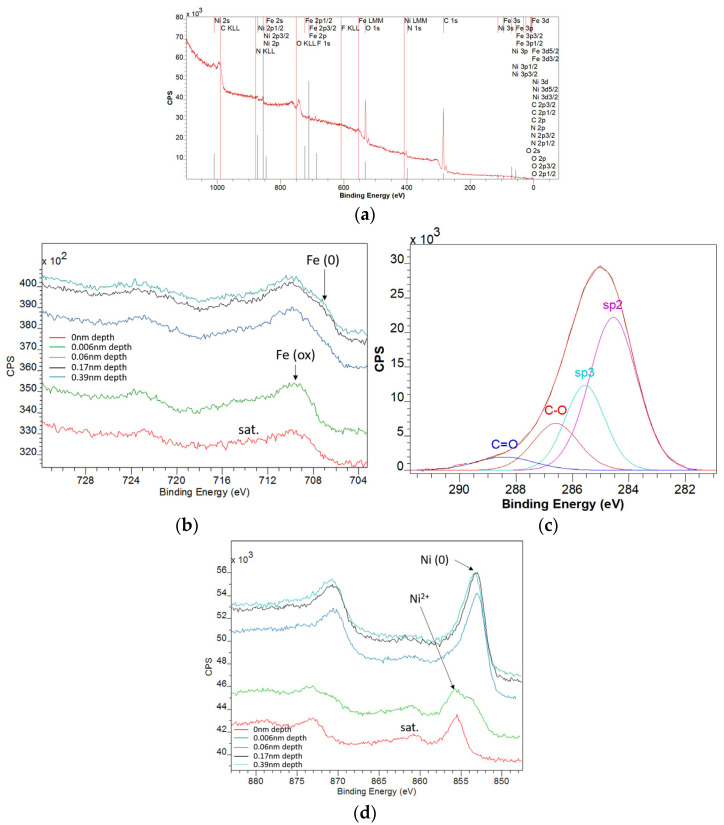
XPS survey spectrum of the GO-Fe_3_O_4_-NiO sample (**a**), XPS spectrum deconvolution corresponding to the Fe 2p line at different depths up to 0.39 nm (**b**), XPS spectrum deconvolution corresponding to the C 1s line (**c**), and XPS spectrum deconvolution corresponding to the Ni 2p line at different depths up to 0.39 nm (**d**).

For the GO-Fe_3_O_4_-NiO sample, from the analysis of the survey spectrum (Figure 8a), it was found that the elements Fe, Ni, F, N, O, and C are present. The analysis of the F 1s line indicated surface contamination with organic fluorine. The multiplet structure of the Fe 2p spectrum (Figure 8b) is characteristic of the 2+ and 3+ oxidation states corresponding to the Fe_3_O_4_ compound. At depths greater than 0.1 nm, the Fe (0) oxidation state appears. At a depth of 0.39 nm, the ratio of Fe(0)/Fe(ox) = 0.2. The analysis of the C1s spectrum (Figure 8c) revealed the specific lines for the graphene oxide with a sp3/sp2 ratio of 0.46. The multiplet structure of the Ni 2p spectrum (Figure 8d) was characteristic of the 2+ and 0 oxidation states. Analysis of the depth profile indicated that the oxidation state of Ni was (0), and there was a superficial oxide layer on the surface. At a depth of 0.39 nm, the ratio of Ni(0)/Ni(ox) = 2.19.

### 3.5. VSM Characterization

**Figure 9:**
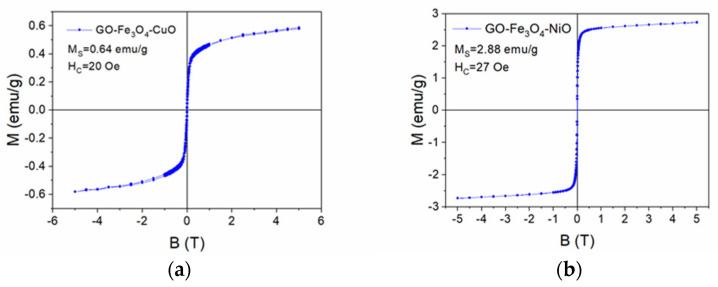
Magnetization as a function of the applied magnetic field for the GO−Fe_3_O_4_−CuO (**a**) and GO−Fe_3_O_4_−NiO samples (**b**).

The magnetization curves vs. the applied magnetic field of the GO-Fe_3_O_4_-CuO and GO-Fe_3_O_4_-NiO samples are presented in Figure 9. The shape of the magnetization curves is typical of superparamagnetic materials. In the case of the GO-Fe_3_O_4_-CuO sample, the saturation magnetization was 0.64 emu/g, and the coercive field was 20 Oe (Figure 9a), while in the case of the GO-Fe_3_O_4_-NiO sample, the saturation magnetization was 2.88 emu/g, and the coercive field was 27 Oe (Figure 9b).

### 3.6. Removal of Pesticides and Drugs from Aqueous Solutions

**Figure 10:**
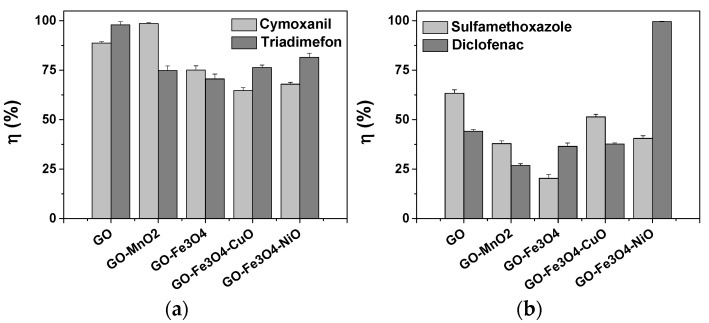
Removal efficiency (η, %) of (**a**) pesticides (cymoxanil and triadimefon) and (**b**) drugs (sulfamethoxazole and diclofenac) from aqueous solution using prepared materials based on graphene oxide (GO) and metal oxides (MOx: MnO_2_, Fe_3_O_4_, CuO, NiO). Tested materials: GO, GO-MnO_2_, GO-Fe_3_O_4_, GO-Fe_3_O_4_-CuO, and GO-Fe_3_O_4_-NiO.

The results of preliminary adsorption tests are promising (Figure 10). The obtained materials presented a better affinity for adsorbing the pesticides rather than the selected drugs. For the pesticides, the removal efficiencies were over 64%. For cymoxanil, the best removal efficiency was obtained using the material GO-MnO_2_ (98.59%), followed by GO (88.77%), GO-Fe_3_O_4_ (74.99%), GO-Fe_3_O_4_-NiO (67.74%), and GO-Fe_3_O_4_-CuO (64.74%) (Figure 10a). The removal efficiencies of triadimefon were 97.93% using the GO material, 81.52% with GO-Fe_3_O_4_-NiO, 76.31% with GO-Fe_3_O_4_-CuO, 74.84% with GO-Mn, and 70.58% with GO-Fe_3_O_4_.

For the selected drugs, the removal efficiencies were lower, between 20.32% and 63.33%. An exception from this trend occurred in the case of diclofenac, with a removal efficiency of 99.59% obtained with the GO-Fe_3_O_4_-NiO material (Figure 10b). Sulfamethoxazole was better adsorbed by the GO materials (63.33%) rather than the other tested materials. After GO, the best materials for sulfamethoxazole removal were GO-Fe_3_O_4_-CuO (51.41%), G-MnO_2_ (37.92%), GO-Fe_3_O_4_-NiO (40.56%), and GO-Fe_3_O_4_ (20.32%). Diclofenac was removed from the aqueous solution in proportions of 26.85% and 99.59% (Figure 10b).

## 4. Conclusions

This research presents the synthesis of graphene oxide (GO)-based materials functionalized with metal oxides (MOx: MnO_2_, Fe_3_O_4_, CuO, NiO), demonstrating their potential applications in water decontamination. The comprehensive characterization using XRD, TEM, FTIR, XPS, and VSM confirmed that the incorporation of the selected MOx significantly modified the morphological, structural, and chemical properties of the GO matrix. The XRD and TEM analyses revealed the successful formation of MOx phases, with noticeable shifts in diffraction patterns and material embeddings. FTIR and XPS spectra highlighted the interaction between GO and the MOx, suggesting possible changes in the electronic structure. The VSM analysis showed that the synthesized materials exhibited superparamagnetic behavior, which may be beneficial for magnetic separation processes in water purification.

The preliminary adsorption tests demonstrated that the synthesized materials showed a higher affinity toward pesticides than the selected drugs. Cymoxanil and triadimefon exhibited high removal efficiencies with G-MnO_2_ (98.59%) and GO (97.93%) materials, which were the most effective materials. Contrarily, the removal of drugs was generally lower, ranging from 20.32% to 63.33%, except for diclofenac, which was removed with high efficiency (99.59%) using the GO-Fe_3_O_4_-NiO material. Sulfamethoxazole showed the best adsorption with GO (63.33%). Overall, these functionalized GO-based materials showed promising prospects for efficient and sustainable water decontamination applications, offering an effective solution to environmental pollution. Therefore, future studies will be focused on the optimization of the adsorption processes for the materials that showed the best affinity for the selected pollutants.

## Data Availability

The original contributions presented in this study are included in the article. Further inquiries can be directed to the corresponding author.
